# Peak velocity estimation in aortic stenosis patients using a fast three-directional two-dimensional phase contrast technique in a single breath-hold: comparison to unidirectional phase contrast MRI and transthoracic echocardiography

**DOI:** 10.1186/1532-429X-18-S1-P335

**Published:** 2016-01-27

**Authors:** Juliana Serafim da Silveira, Matthew E Smyke, Rizwan Ahmad, Ning Jin, Debbie Scandling, Jennifer A Dickerson, Carlos E Rochitte, Subha V Raman, Orlando P Simonetti

**Affiliations:** 1 Department of Internal Medicine/Division of Cardiovascular Medicine, OSU, Columbus, OH USA; 2 Dorothy M. Davis Heart and Lung Research Institute, The Ohio State University, Columbus, OH USA; 3 Siemens Healthcare, Columbus, OH USA; 4 Department of Medicine/Cardiology, InCor Heart Institute, São Paulo, Brazil; 5 Department of Radiology, The Ohio State University, Columbus, OH USA

## Background

**Figure 1:**
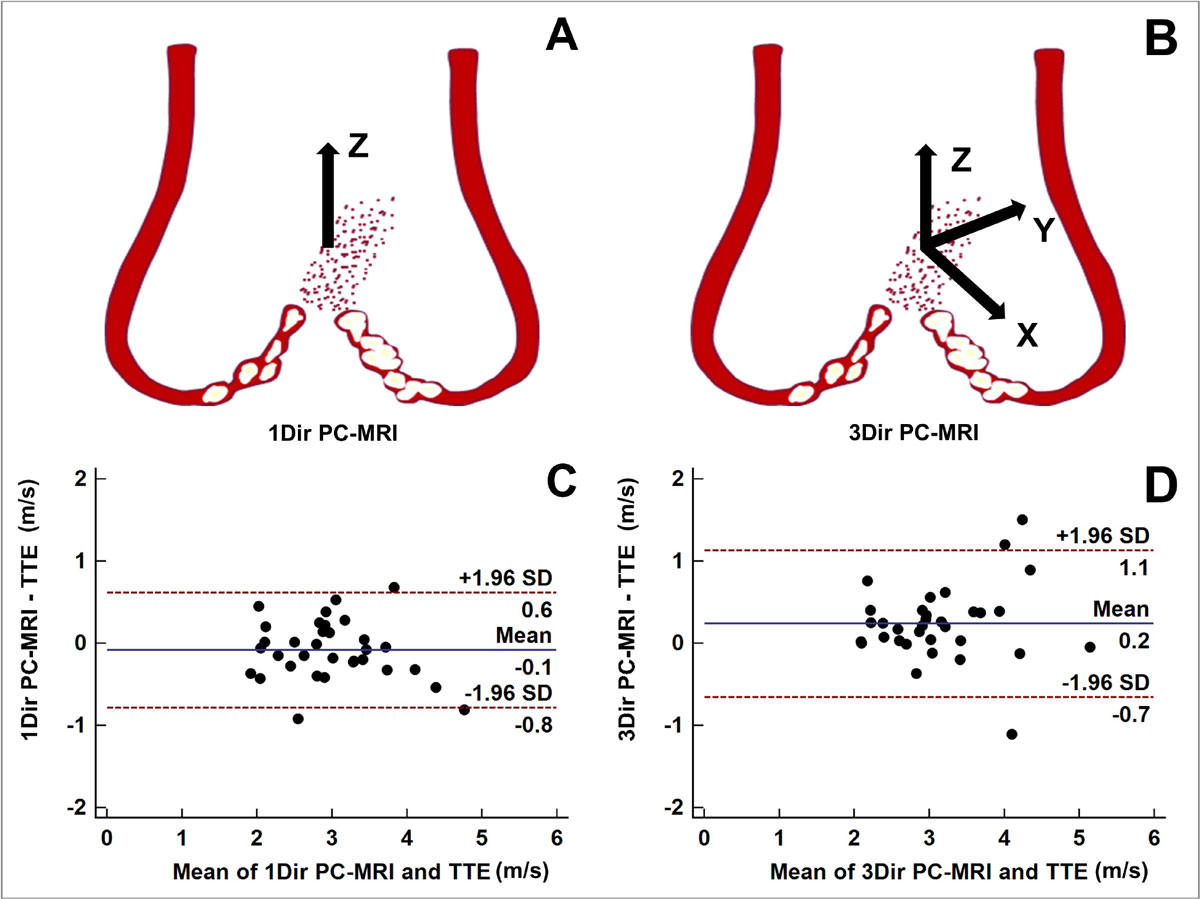
**(A/B) Differences in velocity estimation between 1Dir and 3Dir PC-MRI**. While 1Dir PC-MRI computes velocity in one direction (Z), 3Dir PC-MRI simultaneously computes velocities in 3 directions (X, Y and Z) in a single breath-hold. (C/D) Bland-Altman plots of comparison between peak velocities from TTE versus 1Dir PC-MRI, and TTE versus 3Dir PC-MRI.

Assessment of aortic valve stenosis (AVS) severity is crucial for valve replacement indication and is typically performed by transthoracic Doppler-echocardiography (TTE). However, TTE may be suboptimal in up to 30% of patients. Unidirectional through-plane phase-contrast magnetic resonance imaging (1Dir PC-MRI) is the most common MRI technique used to quantify peak velocities (Vpeak) and flow (Figure 1A). Nonetheless, 1Dir PC-MRI has been shown to underestimate aortic velocities if imaging planes are not prescribed exactly perpendicular to flow direction. Thus, multi-directional velocity quantification would likely improve the accuracy of peak velocity measurements, and allow for more accurate grading of AVS severity. We sought to determine whether a PC technique capable of measuring 3 directions of velocity in a 2D image plane in a single breath-hold (3Dir PC-MRI) (Figure 1B) provides more accurate estimation of Vpeak compared to the traditional 1Dir PC-MRI, using TTE as the reference standard.

## Methods

Patients with variable degrees of aortic valvular disease were prospectively included, and assessed with both TTE and CMR. 1Dir (TR/TE = 49/2.3 ms, α = 250, BW = 420Hz/px, segmented GRE) and 3Dir PC-MRI (TR/TE = 49/2.8 ms, α = 150, BW = 1860 Hz/px, segmented EPI) data were acquired at 3 levels above the aortic valve using a 1.5T Siemens Avanto. Imaging parameters were: 6 mm slice thickness, FOV: 380 × 300 mm^2^, matrix = 192 × 140, Venc 200-550 cm/s, prospective ECG triggering, GRAPPA r = 2. Quantitative image analysis was performed offline using Matlab (Mathworks, Natick, MA). 3Dir PC-MRI Vpeak was calculated pixel by pixel using the root sum square of the three orthogonal velocities (i.e., direction independent speed). After magnitude and flow thresholding to eliminate noise, the pixel with the highest velocity within the valve contour was used for comparison to TTE. Stroke volumes (SV) were also estimated from through-plane 1Dir and 3Dir PC-MRI and compared to left ventricular volumes from SSFP cine imaging.

## Results

Forty-one patients were enrolled (25 males, median age 68 years [range 27-85 years]). The average interval between TTE and CMR was 33 ± 23 days. 1Dir PC-MRI tended to underestimate Vpeak while 3Dir PC-MRI measured a higher Vpeak than TTE. Bland-Altman Plots in Figure 1 C/D illustrate a mean difference of -0.1 m/s and +0.2 m/s for 1Dir and 3Dir PC-MRI, respectively. Good correlation was observed between both 1Dir and 3Dir PC-MRI SV versus cine SV at all levels above the aortic valve (*ρ*_c_ = 0.85 to 0.89), with a slight tendency of SV overestimation by 1Dir PC-MRI and underestimation by 3Dir PC_MRI (Table 1).

**Table 1:** Correlations between 1Dir, 3Dir PC-MRI and SSFP cine imaging stroke volume at different acquisition levels above the aortic valve. A positive bias was observed for 1Dir PC-MRI while a smaller negative bias was observed for 3Dir PC-MRI.

	Plane 0		Plane1		Plane2	
	ρc	Bias ± SD (ml)	ρc	Bias ± SD (ml)	ρc	Bias ± SD (ml)
1Dir PC-MRI	0.85	7 ± 15	0.88	5 ± 12	0.88	4 ± 12
3Dir PC-MRI	0.89	-2 ± 13	0.89	-3 ± 12	0.86	-4 ± 14

ρc: Lin's Concordance Correlation Coefficient

## Conclusions

The higher Vpeak by 3Dir PC-MRI may be explained by its directional independence, as opposed to 1Dir PC-MRI and TTE, which can only accurately measure velocity perpendicular or parallel to the stenotic jet, respectively. 3Dir PC-MRI may therefore offer an advantage over both 1Dir PC-MRI and TTE in the clinical assessment of AVS.

